# Perceived effectiveness of factors and intervention strategies for promoting evidence-based policymaking in Japan: a cross-sectional survey among policymakers, researchers and knowledge brokers

**DOI:** 10.1186/s12961-026-01501-5

**Published:** 2026-07-11

**Authors:** Yuko Arimura, Yuko Yanagawa, Shota Kiuchi, Chikoto Suzuki, Haruka Matsuyama, Hinako Wakabayashi, Hisaya Tomori, Kenji Takehara, Sonoko Sensaki

**Affiliations:** 1 Think Tank for Children and Families, National Center for Child Health and Development, Tokyo, Japan; 2 Department of Medical Technology Implementation and Social Impact , National Center for Child Health and Development, Tokyo, Japan; 3 Department of Social Medicine, National Center for Child Health and Development, Tokyo, Japan; 4 Department of Health Policy, National Center for Child Health and Development, Tokyo, Japan; 5 Department of Psychosocial Medicine, National Center for Child Health and Development, Tokyo, Japan

**Keywords:** Evidence-based policymaking, Policymaker, Researcher, Knowledge brokers

## Abstract

**Background:**

Evidence-based policymaking (EBPM) improves the quality of decision-making and ensures that necessary policies reach the populations who need them. Although EBPM-promoting factors have been conceptually explored, interventions that comprehensively address these factors remain under-investigated. Through qualitative research, we previously identified 25 factors that promote EBPM. Thus, this study aimed to (1) evaluate the perceived effectiveness of factors that promote EBPM and (2) assess the perceived effectiveness of interventions designed to address these factors.

**Methods:**

A cross-sectional survey was conducted in Japan among three stakeholder groups: policymakers, researchers and knowledge brokers (KBs). Participants assessed the perceived effectiveness of the 25 factors and 75 corresponding intervention strategies. The mean scores were calculated for each factor, and the proportion of respondents who considered each intervention effective within its respective factor was determined.

**Results:**

Of the 154 invited participants, 55 completed the survey, including policymakers (*n* = 15), researchers (*n* = 25) and KBs (*n* = 15). The highest-rated factor was “organizing and disseminating leverageable evidence and data” (mean = 4.53), which included the intervention “development of data infrastructure easily utilizable by researchers while ensuring personal information protection”, selected as effective by 78.2% of the respondents. The second highest-rated factor was “enhancing the knowledge and skills of policymakers and researchers in policymaking processes, EBPM, and research” (mean = 4.37). Within this factor, two interventions shared the highest selection rate (72.3%): “establishment of an in-ministry training system enabling policymakers to acquire essential skills regardless of department” and “development and provision of EBPM-related educational content for professional organizations (academic societies) and policymakers”.

**Conclusions:**

This study identified the perceived effectiveness of 25 EBPM-promoting factors and 75 related interventions in the Japanese policymaking context on the basis of input from policymakers, researchers and KBs. These findings provide a foundation for the implementation of strategies by outlining practical interventions. The descriptive trends in perceived effectiveness across stakeholders suggest that implementation should reflect each group’s priorities, roles and constraints. This study contributes to EBPM literature by systematically linking factors with interventions and demonstrating how stakeholder perspectives differ, thereby strengthening the basis for promoting EBPM in practice.

**Supplementary Information:**

The online version contains supplementary material available at https://doi.org/10.1186/s12961-026-01501-5.

## Background

Evidence-based policymaking (EBPM) has the potential to strengthen the use of evidence during policy formulation [1–4]. However, in the decision-making process, evidence is often weighed against competing external factors, such as political pressure, fiscal limitations and legal constraints [1, 5]. Thus, while EBPM can support higher-quality information-gathering for decision-making, it does not necessarily guarantee the final outcomes, as evidence may be marginalized by political considerations [5, 6]. Furthermore, the use of evidence in policy formulation aims to ensure that the needs of target populations are more rigorously accounted for, though this does not automatically ensure successful policy delivery or reach [7]. Several EBPM-related factors influence the integration of evidence, including collaboration, organizational resources and the characteristics of researchers and policymakers [1, 8, 9]. Promoting EBPM requires increasing facilitators while reducing barriers, acknowledging that different stakeholders may prioritize different types of evidence depending on their roles in the policymaking process [7]. In Japan, EBPM has been promoted as a national initiative since 2017; however, its implementation continues to face challenges similar to those observed internationally [10, 11].

Various interventions have been implemented to address the individual factors that promote EBPM [12]. For instance, knowledge brokering has been explored as a strategy to enhance contact and collaboration between stakeholders [13]. Arimura et al. investigated EBPM in Japan and comprehensively identified factors that facilitate its promotion [14]. However, interventions that incorporate these factors have not yet been investigated. To address this gap, this study developed 75 interventions linked to 25 previously identified factors [14], designed to support their efficient implementation within existing resource constraints. Establishing priorities and implementing appropriate interventions are therefore essential.

To maximize the use of limited resources, interventions should be implemented on the basis of priority. Health policy frameworks highlight the critical role of priority setting in effective decision-making. In line with this context, the Evidence-Informed Policy Network, established by the World Health Organization, assists countries in systematically integrating research evidence into policy decisions and recommends prioritizing evidence-based implementation [15]. Moreover, various criteria for priority setting have been identified. Similarly, the Value- and Evidence-Based Decision Making and Practice framework assesses the value of evidence, such as efficacy, acceptability, cost–effectiveness and political will. This highlights the importance of balancing empirical strength and policy feasibility [16]. Accordingly, in this study, we prioritized factors and interventions by estimating their value on the basis of the stakeholders’ perceived effectiveness of each intervention.

Therefore, this study aimed to (1) evaluate the perceived effectiveness of factors that promote EBPM and (2) assess the perceived effectiveness of interventions designed to address these factors within the Japanese policymaking context.

## Methods

### Study design

This study builds on a previous study that identified 25 factors that promote EBPM in Japan [14]. This study further examined the perceived effectiveness of 25 factors and their corresponding intervention strategies. A cross-sectional survey using an anonymous, self-administered web-based questionnaire was conducted between 1 October 2024 and 15 November 2024. The target population included individuals involved in implementing EBPM in the field of maternal and child healthcare policies in Japan, including policymakers, researchers and knowledge brokers (KBs). KBs function as human agents who facilitate the transfer of knowledge and bridge the gap between research evidence and policymaking, thereby promoting EBPM [17–19]. The Strengthening the Reporting of Observational Studies in Epidemiology Statement for cross-sectional studies was used to guide the reporting of this study and enhance its quality and transparency [20].

### Developing the questionnaire

A questionnaire was developed on the basis of a semi-structured interview conducted previously [14]. The interview identified five categories and 25 factors that promoted EBPM. These factors were used to assess the perceived effectiveness. Intervention strategies were developed for each of the 25 factors through a literature review and discussion by YA, SK, YY, HM, KT, and SS. After finalizing the intervention strategies within the research team, multiple researchers outside the team were consulted to verify the completeness and appropriateness of the content in relation to the study objectives and the Japanese policymaking context. Ultimately, 75 intervention strategies were included to assess perceived effectiveness.

### Selecting and recruiting the participants

Because this study aimed to examine the perceived effectiveness of factors and interventions that could advance EBPM, a highly specialized policy domain, participants with relevant expertise and experience were recruited. Consequently, the number of eligible experts from both inside and outside government was inherently limited. Potential participants from each stakeholder group were identified and recruited based on the following criteria:

1. Study participants could be policymakers currently or formerly affiliated with governmental authorities within the past 5 years, with ministries related to child and maternal health policies. Policymakers commonly rotate across departments every 2–3 years. Throughout these rotations, they are tasked with setting policy agendas, identifying emerging policy needs, and formulating and evaluating policy measures. YA, YY, HT, HM, and SS approached the relevant departments of each ministry to explain the purpose of the study and asked them to support the recruitment process. The target participants were invited to participate via emails sent by their respective departments.
2. Study participants could be researchers who, within the past 5 years, have served as principal investigators of the Health Labour Sciences Research Grant funded by the Ministry of Health, Labour and Welfare. Policymakers are also involved in generating evidence by commissioning research projects, including those supported by the Ministry of Health, Labour and Welfare. In addition, some researchers are appointed as external experts to national councils and advisory committees, where they contribute academic perspectives on specific policy areas. However, such appointments remain limited and not widely accessible to the broader research community. SK compiled a list based on publicly available information from the Health Labour Sciences Research Grant databases. For individuals whose contact information was not publicly available, we first obtained their consent to provide personal information and then acquired their contact details through the research team’s network. After obtaining personal information, SS contacted them to explain the purpose of the study and requested their cooperation in the survey via email or postal mail.
3. Study participants could be KBs who have previously worked as technical officers through personnel exchanges within ministries related to child and maternal health policies. Japan operates a personnel exchange system in which professionals from private and healthcare sectors, including hospitals, are seconded to ministries, research institutes or clinical settings. These individuals are expected to act as KBs, facilitating connections between health policies, practice settings, such as local governments and clinical fields, and scientific evidence. These secondments typically last for 2 years, although extensions are possible. YA, YY, HT, HM, and SS approached the relevant departments of each ministry to explain the purpose of the study. The target participants were invited to participate via emails sent by their respective departments. For individuals who had previously participated in personnel exchanges and were currently affiliated with other institutions, we first obtained their consent to provide personal information and acquired their contact details on Microsoft Forms. Then, SS contacted them to explain the purpose of the study and requested their cooperation in the survey via email.

### Data collection

An anonymous, self-administered survey collected the following data: (1) basic attributes of the respondents, including current affiliation, years of working experience in government agencies, research institutions and personnel exchanges; (2) perceived effectiveness of each 25 factors on a five-point Likert scale (very effective, effective, neutral, not effective and not effective at all) (participants who did not understand the question could select “do not understand”); and (3) perceived effectiveness of 75 intervention strategies, with participants selecting all strategies they consider effective within each category. The survey was administered using Microsoft Forms, which does not require respondents to provide personal information, including names.

### Data analysis

Descriptive analyses were performed to summarize the basic attributes of the respondents. The mean scores were calculated for the perceived effectiveness of each factor. In addition, the proportion of respondents who considered each intervention to be effective within its respective factors was calculated.

## Results

In total, 154 participants, including policymakers (*n* = 78), researchers (*n* = 49) and KBs (*n* = 27), were invited to participate in this study. Of the 154 participants, 55 completed the survey. Response rates varied across stakeholders: 51.0% among researchers, 55.6% among KBs and 19.2% among policymakers. The average years of experience for policymakers were 9.1 (range, 2–19) years, whereas for researchers, it was 23.5 (range, 5–41) years. The average duration of personnel exchange for KBs was 3.2 (range, 0.5–7) years (Table 1).

**Table 1:** Demographics of the study participants

	Policymaker		Researcher		Knowledge brokers	
	*n*, mean	(%, range)	*n*, mean	(%, range)	*n*, mean	(%, range)
Number of target participants	78	–	49	–	27	–
Number of respondents	15	(19.2)	25	(51.0)	15	(55.6)
Years of experience (range)^*^	9.1	(2–19)	23.6	(5- 41)	3.2	(0.5- 7)

*The years of experience for KB indicate the total number of personnel exchanges

### Perceived effectiveness of factors

Among the 25 factors identified through the preceding qualitative study [14], the mean perceived effectiveness scores ranged from 3.91 to 4.53, with an overall average of 4.22 (Table 2). In total, 12 factors had mean scores higher than the overall average. Among them, the relatively highly rated factors included the following: “organizing and disseminating leverageable evidence and data” (mean score = 4.53); “enhancing the knowledge and skills of policymakers and researchers in policymaking processes, EBPM and research” (4.37); “securing sufficient budgets, personnel, and time within ministries” (4.36); “evaluating the current situation based on accurate data and qualitatively collected field insights” (4.35); and “developing flexible frameworks for adjusting policy trajectories” (4.35).

**Table 2:** Factors and average scores for EBPM promotion, ordered on the basis of average scores in descending order

Ranking	Factors	All
1	Organizing and disseminating leverageable evidence and data	4.53
2	Enhancing the knowledge and skills of policymakers and researchers in policymaking processes, EBPM and research	4.37
3	Ensuring appropriate allocation of budgetary, personnel and time resources within the government	4.36
4	Evaluating the current situation based on accurate data and qualitatively collected field insights	4.35
4	Developing flexible frameworks for adjusting policy trajectories	4.35
6	Continuing involvement in policymaking from external organizations with specialized knowledge and field expertise	4.29
7	Creating support systems to provide technical and psychological assistance to personnel exchange participants	4.28
8	Establishing systems that can fairly reference evidence and expert opinions	4.27
9	Improving the research environment in academia to encourage research that contributes to policies	4.26
10	Generating personnel exchange participants by providing lectures and hands-on experience and establishing human resource development systems	4.23
11	Introducing schemes to strengthen implementation capabilities at the local government level	4.22
11	Providing organizational support to utilize the experiences of personnel exchange participants	4.22
11	Promoting shared inter-organizational awareness throughout the EBPM and at each stage	4.22
14	Setting agendas from a comprehensive perspective	4.21
15	Implementing administrative procedures that reflect not only central government ministries and agencies but also external organizational perspectives in policy evaluation and effectively utilize the outcomes	4.20
15	Introducing career opportunities for personnel exchange participants after their exchange programmes	4.20
15	Ensuring the thorough dissemination of policies and information to local governments and policy beneficiaries	4.20
18	Establishing and utilizing think tanks to provide scientifically reliable information	4.19
19	Creating platforms for active exchange of opinions and relationships building across organizational boundaries	4.18
19	Generating flexible evidence by researchers meeting the policy needs and establishing administrative mechanisms to leverage them	4.18
21	Citizen participatory policy formation	4.15
21	Increasing awareness and establishing evaluation systems for policies within the government	4.15
23	Constructing personnel evaluation systems considering their impact on policies in academia	4.00
24	Instituting administrative personnel systems that enable long-term engagement in policies	3.98
25	Establishing personnel evaluation systems considering the policy implementation performance of policymakers and policy evaluations	3.91
	Average	4.22

The following three factors had mean scores equal to the overall average (4.22): “introducing schemes to strengthen implementation capabilities at the local government level”, “providing organizational support to utilize the experiences of personnel exchange participants”, and “promoting shared inter-organizational awareness throughout the EBPM and at each stage”.

Conversely, 12 factors had mean scores below the overall average. Among them, the relatively lower scored factors were as follows: “constructing personnel evaluation systems considering their impact on policies in academia” (4.00), “instituting administrative personnel systems that enable long-term engagement in policies” (3.98) and “establishing personnel evaluation systems considering the policy implementation performance of policymakers and policy evaluations” (3.91).

### Variations in stakeholder perceptions and priorities

Table 3 presents the comparison of the top 10 priority factors across stakeholders. A common priority identified across all three stakeholder groups was “organizing and disseminating leverageable evidence and data”.

**Table 3:**
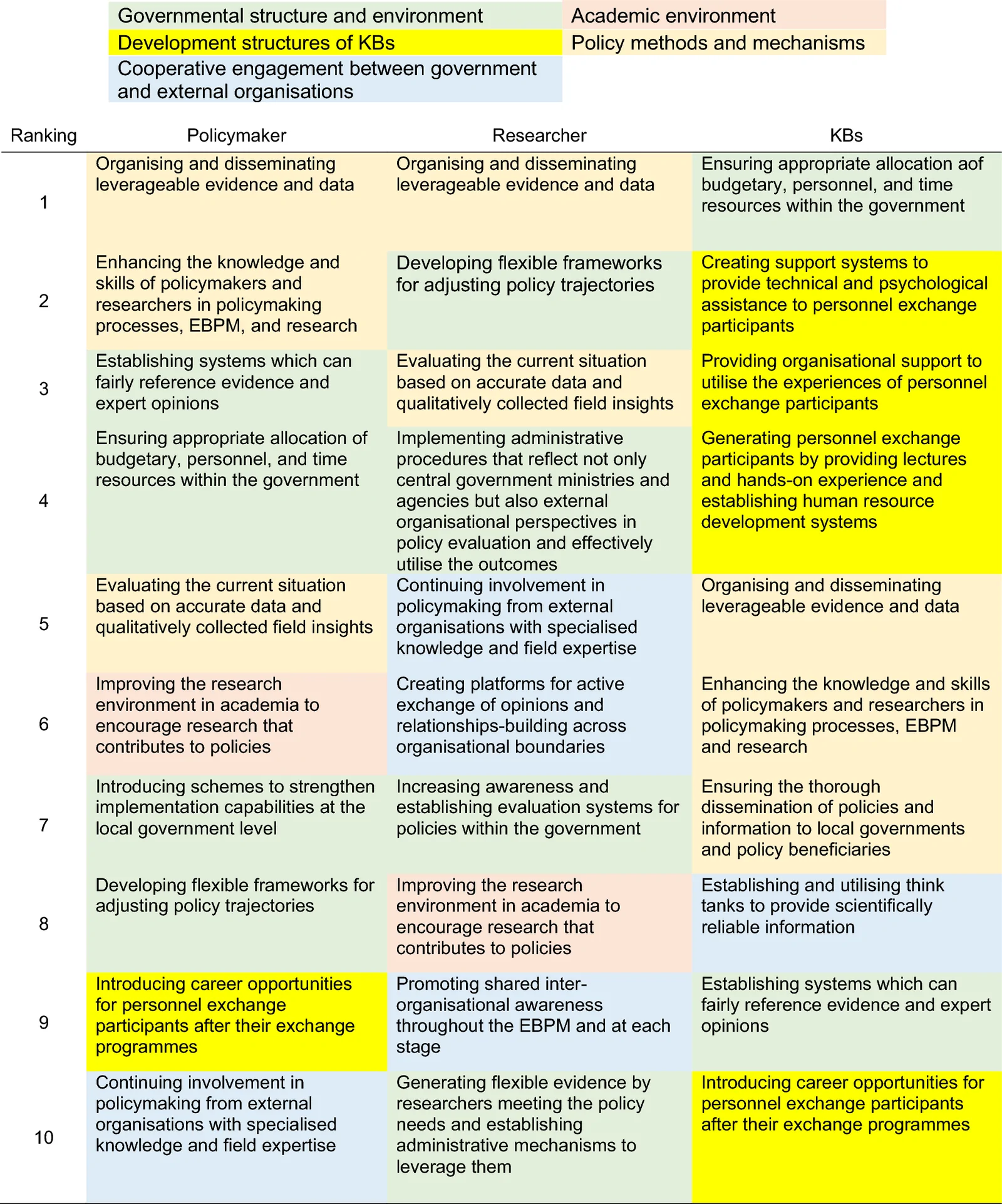
Factors with average scores above the mean and their corresponding interventions

The following sections detail the variation trends in prioritization observed among each stakeholder group. For the policymaker group, the highest-ranked factors were predominantly associated with policy methods and governmental structure and environment. Specifically, key priorities included “organizing and disseminating leverageable evidence and data” and “enhancing the knowledge and skills of policymakers and researchers in policymaking processes, EBPM and research”. Furthermore, institutional factors within the government, such as “establishing systems which can fairly reference evidence and expert opinions” and “ensuring appropriate allocation of budgetary, personnel and time resources within the government”, were among the top positions.

In contrast, factors related to collaboration were noticeably less prioritized by this group. Only one factor from the collaboration category, “continuing involvement in policymaking from external organizations with specialized knowledge and field expertise”, was ranked within their top 10.

Researchers were primarily split between collaboration and the governmental structure and environment. However, a more detailed examination reveals that all top-ranked factors within the governmental category specifically concerned policy evaluation and subsequent policy adjustments. Furthermore, the researcher group did not rank any factors related to KBs within their top 10. Instead, their priorities included the improvement of their own research environments, with one factor specifically addressing this need.

Priorities of the KBs were centred primarily on the development of KBs and the governmental structure and environment. Notably, the factor ‘ensuring appropriate allocation of budgetary, personnel and time resources within the government’ was identified as their highest priority, suggesting it is perceived as the most critical bottleneck for EBPM from an intermediary perspective. Furthermore, KBs highlighted the necessity of improving policy methods, specifically through “organizing and disseminating leverageable evidence and data” and “enhancing the knowledge and skills of policymakers and researchers in policymaking processes, EBPM and research”. In contrast, no factors related to the academic environment were ranked within their top 10.

### Corresponding interventions

Table 4 presents all the interventions corresponding to factors scored above the overall average. In the main text, the intervention with the highest average selection rate for each factor is described below.

**Table 4:** Factors with average scores above the mean and their corresponding interventions

Rank	Factors	Average				Intervention strategies	Average
		All	Policymaker	Researcher	KBs		All
1	Organizing and disseminating leverageable evidence and data	4.53	4.60	4.56	4.40	Development of data infrastructure that researchers can easily utilize while ensuring considerations for personal information	79.6
						Creation and dissemination of data by researchers that consider policy needs	68.5
						Establishment of standardized policy evaluation indicators for local governments and corresponding data collection frameworks	61.1
						Development of a data collection and publication system by ministries to enable policy impact evaluations by external organizations	51.9
2	Enhancing the knowledge and skills of policymakers and researchers in policymaking processes, EBPM and research	4.37	4.40	4.33	4.40	Development and provision of EBPM-related educational content for professional organizations and policymakers	75.5
						Establishment of an in-ministry training system that enables policymakers to acquire essential skills regardless of their assigned department	75.5
						Provision of training materials to new members of ministry committees to facilitate an understanding of administrative systems and policy processes	56.6
3	Ensuring appropriate allocation of budgetary, personnel and time resources within the government	4.36	4.40	4.24	4.53	Implementation of operational efficiency improvements through IT tools	75.5
						Public disclosure of parliamentarians responsible for delayed parliamentary question notices	58.5
						Relaxation of recruitment criteria to increase mid-career hires	43.4
4	Evaluating the current situation based on accurate data and qualitatively collected field insights	4.35	4.33	4.48	4.13	Establishment of forums for policy research experts and policymakers to regularly monitor domestic and international trends and practical realities	77.8
						Establishment of a system within ministries to conduct high-quality research	68.5
						Establishment of external organizations capable of promptly collecting input from children, stakeholders and frontline professionals	51.9
4	Developing flexible frameworks for adjusting policy trajectories	4.35	4.27	4.52	4.13	Establishment of platforms where policymakers and local government officials can discuss policies	88.7
6	Continuing involvement in policymaking from external organizations with specialized knowledge and field expertise	4.29	4.13	4.44	4.20	Creation of a system that allows personnel exchange participants to remain engaged with the policies they worked on during their exchange period	63.6
						Placement of researchers within ministries to conduct research on confidential administrative data	50.9
						Public communication by external organizations on issues difficult for ministries to directly address	40.0
7	Creating support systems to provide technical and psychological assistance to personnel exchange participants	4.28	4.13	4.25	4.47	Creating a guide to provide personnel exchange candidates and host organizations with the necessary knowledge prior to commencement	87.0
8	Establishing systems that can fairly reference evidence and expert opinions	4.27	4.40	4.20	4.27	Development of a system enabling policymakers to refer to scientific knowledge and academic research when assessing current conditions and setting policy agendas	83.3
						Creation of a system allowing policymakers to consult experts when necessary	81.5
9	Improving the research environment in academia to encourage research that contributes to policies	4.26	4.29	4.38	4.07	Enhancement of public statistical information infrastructure to facilitate its utilization by academia	86.3
						Expansion of research funding	80.4
						Establishment of networks among researchers	62.7
						Establishment of a researcher evaluation system that recognizes publications related to policy impact assessment	41.2
10	Generating personnel exchange participants by providing lectures and hands-on experience and establishing human resource development systems	4.23	4.07	4.21	4.40	Development of a system allowing personnel exchange participants to engage in bidirectional interactions with government agencies, academia and clinical settings to learn from diverse perspectives and roles	82.7
						Strengthening of public health workforce development through the enhancement of healthcare policy and public health curricula in public health graduate schools and medical education	73.1
						Provision of practical support for personnel exchange participants by external organizations	42.3
11	Introducing schemes to strengthen implementation capabilities at the local government level	4.22	4.29	4.32	4.00	Strengthening of systems enabling local governments to share and reference best practices	73.1
						Development of training materials by external organizations to enhance EBPM-related skills among professionals, such as public health nurses and midwives	51.9
						Facilitation by external organizations to connect local governments with private sector and academic institutions on the basis of their policy needs	51.9
						Design and implementation of model projects	50.0
						Support for effective and efficient policy implementation by external organizations	46.2
						Implementation of industry–government–academia–civil society collaboration support programmes	36.5
						Government support for the establishment of EBPM Promotion Offices within local governments	34.6
11	Providing organizational support to utilize the experiences of personnel exchange participants	4.22	4.07	4.17	4.47	Establishment of a personnel system in the dispatching organizations to enable personnel exchange participants to leverage their administrative experience post-exchange	75.9
						Establishment of platforms where personnel exchange participants can act as bridges between academia and policy	72.2
						Provision of posts where personnel exchange participants can apply their experiences	70.4
						Development of a system where personnel exchange participants can provide insights to researchers during policy-related research agenda setting	55.6
11	Promoting shared inter-organizational awareness throughout the EBPM and at each stage	4.22	4.13	4.36	4.07	Distribution of training materials by external organizations to facilitate learning on EBPM, policy processes and research fundamentals for both policymakers and researchers	71.7

The factor with the highest mean score, “organizing and disseminating leverageable evidence and data”, included the intervention “development of data infrastructure that researchers can easily utilize while ensuring considerations for personal information”, which had the highest selection rate at 79.6%. For the factor “enhancing the knowledge and skills of policymakers and researchers in policymaking processes, EBPM and research”, two interventions shared the highest selection rate of 75.5%: “establishment of an in-ministry training system that enables policymakers to acquire essential skills regardless of their assigned department” and “development and provision of EBPM-related educational content for professional organizations (academic societies) and policymakers”. In the third-ranked factor, “securing sufficient budgets, personnel and time within ministries”, the intervention with the highest selection rate was the “implementation of operational efficiency improvements through Information Technology (IT) tools”, selected by 75.5% of the respondents.

For the factor “evaluating the current situation based on accurate data and qualitatively collected field insights”, the most highly rated intervention was the “establishment of forums for policy research experts and policymakers to regularly monitor domestic and international trends and practical realities”, with a selection rate of 77.8%. For the factor “developing flexible frameworks for adjusting policy trajectories”, the intervention with the highest selection rate was “establishment of platforms where policymakers and local government officials can discuss policies”, which was selected by 88.7% of the respondents. In the factor “continuing involvement in policymaking from external organizations with specialized knowledge and field expertise”, the most selected intervention was “creation of a system that allows personnel exchange participants to remain engaged with the policies they worked on during their exchange period” (63.6%). For the factor “creating support systems to provide technical and psychological assistance to personnel exchange participants”, the intervention “creating a guide to provide personnel exchange candidates and host organizations with the necessary knowledge prior to commencement” received a high selection rate of 87.0%.

In the eighth-ranked factor, “establishing systems that can fairly reference evidence and expert opinions”, the intervention “development of a system enabling policymakers to refer to scientific knowledge and academic research when assessing current conditions and setting policy agendas”, had a selection rate of 83.3%. For the factor “improving the research environment in academia to encourage research that contributes to policies”, the highest-rated intervention was “enhancement of public statistical information infrastructure to facilitate its utilization by academia”, which was supported by 86.3% of the respondents. Regarding the factor “generating personnel exchange participants by providing lectures and hands-on experience and establishing human resource development systems”, 82.7% of the respondents selected “development of a system allowing personnel exchange participants to engage in bidirectional interactions with government agencies, academia and clinical settings to learn from diverse perspectives and roles”.

For the factor ranked eleventh, “introducing schemes to strengthen implementation capabilities at the local government level”, the highest-rated intervention was “strengthening systems enabling local governments to share and reference best practices”, with a selection rate of 73.1%. In the equally ranked factor “providing organizational support to utilize the experiences of personnel exchange participants”, the intervention “establishment of a personnel system in the dispatching organizations to enable personnel exchange participants to leverage their administrative experience post-exchange” received the highest selection rate (75.9%). Finally, in the factor “promoting shared inter-organizational awareness throughout the EBPM and at each stage”, the highest-rated intervention was “distribution of training materials by external organizations to facilitate learning on EBPM, policy processes and research fundamentals for both policymakers and researchers”, with a selection rate of 71.7%.

## Discussion

### Summary of findings

Building on a previous qualitative study [14] that identified five categories and 25 factors critical for promoting EBPM among policymakers, researchers and KBs in Japan, this study quantitatively assessed the perceived effectiveness of each factor. It further evaluated the perceived effectiveness of specific, implementable interventions aligned with each factor, thereby offering practical implications for the development and implementation of strategies to advance EBPM.

### Key features of factors perceived as effective for promoting evidence-based policymaking

Among the top five highest-rated factors, relatively few were related to “assessing the current situations” and “policy evaluation”, which are key phases of the EBPM cycle [21, 22] (Table 2). This underscores the importance of grounding policy decisions in terms of accuracy, understanding the field and maintaining institutional mechanisms for ongoing policy evaluation. Previous qualitative findings [14] have pointed out that the challenges and needs on the ground are often recognized through public opinion or petitions, rather than scientific evidence. Therefore, it is essential to combine quantitative and qualitative approaches to capture field reality. In addition, the lack of policy evaluation is a recognized issue not only in Japan but also in many other countries. Even in the United Kingdom, which is often regarded as an advanced country in EBPM, the implementation of policy evaluations remains limited, despite the availability of toolkits, such as the Magenta Book to guide practices [23]. Furthermore, factors related to securing organizational resources, including personnel, time and budget, and improving individual EBPM-related competencies within ministries were among the top five factors. These findings reaffirm that advancing EBPM requires both institutional capacity in ministries and human capital development responsible for EBPM execution.

### Divergent priorities and underlying reasons across stakeholders

The descriptive analysis of stakeholder priorities revealed three key thematic trends, which reflect the unique institutional contexts and professional constraints of each stakeholder. A common priority identified across all three stakeholder groups was “organizing and disseminating leverageable evidence and data”. This shared consensus suggests that, despite their differing professional perspectives, all actors perceive the lack of accessible and actionable data as a primary systemic barrier. It further indicates that establishing a robust data infrastructure is recognized not solely as a technical requirement but also as a fundamental prerequisite for advancing EBPM. In contrast, the analysis highlighted notable divergences in priorities among the three stakeholder groups. The following sections explore these divergences in detail, focusing on the specific constraints and expectations of each stakeholder category.

#### Disparity in motivation for collaboration

The findings reveal a clear disparity in motivation for collaboration between researchers and policymakers. While our previous qualitative research [14] indicated that policymakers recognize the intrinsic value of dialogue—noting that it is “very important to have places where policymakers and researchers can speak openly and listen to each other”—researchers similarly emphasized the need for conceptual alignment, stating, “Even when we use the same word, like ‘evidence’, what policymakers consider evidence often differs from what researchers mean… It is important to have a shared understanding between researchers and the government about what constitutes sufficient consensus” [14]. However, these current quantitative results indicate that researchers express a substantially stronger inclination towards actual engagement. This suggests that policymakers prioritize internal structural and methodological readiness takes precedence over external partnerships. Such hesitation is likely rooted in acute practical barriers, specifically a chronic lack of time, personnel and budgetary resources. Prior research suggests that the administrative burden often outweighs the capacity for external engagement [24]. Consequently, policymakers appear focused on administrative consolidation and immediate resource allocation, leaving them with limited capacity to initiate the cross-sectoral collaborations desired by researchers.

#### Differing perceptions of KBs

There is a notable trend in how stakeholders perceive the importance and professional benefits of KB roles. While policymakers and KBs themselves recognize the necessity of these intermediary functions to bridge the gap between evidence and action, researchers ranked KB-related factors relatively lower. This gap in recognition may be attributed to a structural misalignment between academic achievements and EBPM activities. As our previous study found, “we need to think about career paths that include personnel exchanges and adjust the evaluation system on the academic side” [14]. Currently, institutional evaluation systems in academia do not fully reward cross-sectoral exchange as a formal performance metric, leading researchers to perceive fewer direct professional benefits from brokerage roles. Without addressing these institutional gaps, as prior research suggested, the promotion of EBPM will remain hindered by a lack of organizational practices that incentivize brokerage [7].

#### Step-specific focus within the EBPM process

Stakeholder perceptions tend to be concentrated on the specific stages of the policy cycle they directly handle, rather than the entire EBPM process, which encompasses problem identification, policy formulation, decision-making, implementation and evaluation [25, 26]. Policymakers focus predominantly on the implementation necessary to sustain policy tasks, whereas researchers prioritize the beginnings and ends of the cycle, specifically problem identification and policy evaluation, which aligns with their professional expertise in data analysis. In contrast, KBs, viewing the system from an intermediary perspective, identified the establishment and utilization of third-party organizations such as think tanks to provide scientifically reliable information as the primary bottleneck for the entire process. This stage-specific focus suggests that each group operates within its own institutional boundaries, leading to a fragmented approach to the policy cycle. Prior research indicates that the mere existence of data does not guarantee its effective use [24]; instead, it requires a structural capacity to process evidence consistently across all stages of the cycle. Our findings emphasize that the stakeholders must engage beyond their immediate departmental roles and work across institutional boundaries for EBPM to function as a continuous and integrated cycle, rather than a series of transient activities.

### Key features of interventions perceived as effective for promoting EBPM

We categorized the intervention strategies into four major groups, which were perceived as effective by > 70% of the respondents across all stakeholders. Although this study did not use the Delphi method, a threshold of 70% was adopted with reference to the consensus criteria commonly used in Delphi research [27]. The four major categories are as follows: (1) creating an environment that fosters innovation and evidence generation, (2) establishing sustainable cross-sectoral partnerships, (3) enhancing EBPM-related skills across stakeholders, and (4) providing structured support for KBs. These domains represent the strategic priorities that can effectively promote the advancement of EBPM. The following paragraphs provide detailed discussions of each item:

1. Creating an environment that supports commitment to evidence generation.

This domain encompasses the development of systems and resources that support the generation, accessibility and utilization of evidence. The suggested approaches include developing data and statistical systems that are accessible to researchers, considering personal information, promoting administrative operations by utilizing information technology (IT) tools and expanding financial support for research activities. Prior research [1] highlighted challenges, such as limited access to structured public data and persistent resource constraints within government agencies. In line with this, respondents emphasized the need to enhance the accessibility of well-organized public data and utilize IT tools to address ongoing shortages in capacity. These findings are consistent with the current national efforts toward medical and administrative digital transformation in Japan [28].

2. Establishing sustainable stakeholder partnerships.

This domain involves establishing mechanisms that facilitate sustained collaboration among policymakers, local government officials, researchers and other relevant stakeholders. These include efforts to create platforms for intergovernmental dialogue, strengthen systems that enable EBPM through access to expert knowledge and scientific evidence, promote mutual learning through the exchange of best practices and local innovations among municipalities and strengthen the use of scientific knowledge for problem identification and policymaking. Previous literature [29] has indicated a fragmentation between academic and policy communities, highlighting the need for structures that enable regular interaction. In Japan’s health sector, particularly in maternal and child health, local governments play a central role in policy implementation. However, qualitative findings from our previous study [14] suggest that the experiences and challenges of municipalities are often insufficiently shared with ministries, resulting in policies that lack practical adaptability. Establishing platforms for continuous, bidirectional dialogue can foster shared understanding by enabling mutual learning and diffusion of effective practices.

3. Enhancing EBPM-related individual skills.

This area encompasses the development and dissemination of educational resources that enhance stakeholders’ competencies related to EBPM. It includes the provision of EBPM-related training content, the distribution of accessible learning materials by external organizations to support the understanding of public policy processes and scientific research, the establishment of internal training systems within ministries to ensure consistent skill development regardless of departmental rotations and the strengthening of academic curricula in schools of public health and medical schools in the fields of health policy and public health to enhance the development of human resources in public health. Prior studies [1, 9, 14, 30] have shown that both researchers and policymakers are often aware of their own knowledge gaps in relation to the policymaking process and evidence generation. Our previous work [14] highlighted that researchers’ lack of familiarity with the policymaking process often prevented them from supplying timely and actionable research evidence. Therefore, the development and dissemination of accessible learning opportunities and foundational resources designed to support EBPM is not only beneficial but also essential to addressing this gap.

4. Providing support for knowledge brokers.

This component encompasses the development of institutional frameworks and practical resources to support KBs in their role as a bridge between evidence and policies. Potential approaches include establishing systems that facilitate cross-sectoral personnel exchanges; providing opportunities for KBs to acquire diverse perspectives through interactions with government, academia and clinical settings; ensuring that home institutions recognize and make use of skills acquired through secondments; and developing preparatory guidance materials to assist both home and host organizations in navigating the transition process. The importance of KBs in facilitating evidence has been demonstrated in previous studies [18, 19, 31]. Nevertheless, our previous study [14] revealed insufficient institutional mechanisms in Japan to support KBs effectively throughout the personnel exchange process. Among the proposed measures, the development of practical orientation manuals with clear and accessible content emerged as one of the most frequently endorsed strategies. These materials require relatively limited institutional investment, are adaptable to diverse organizational contexts and appear to be readily disseminated across institutions. In addition to these resources, establishing structured peer-learning networks may be highly beneficial, as the specialized nature of the KB role can often lead to professional isolation. Although personnel exchanges are typically conducted over a period of 2 years, implementing them over a shorter duration may also prove beneficial. Even relatively brief exchanges can enable KBs to develop a thorough understanding of the different perspectives and operational conditions across sectors. A notable international example is the US Intergovernmental Personnel Act, which provides a formal legal framework for facilitating exchanges between federal and non-federal entities [32]. This suggests that adopting similarly structured exchange mechanisms in other contexts can enhance knowledge transfer and strengthen the collaborative capacity among stakeholders in EBPM.

### Strengths and limitations

This study has some limitations. First, the response rates differed across stakeholders: approximately 50% of researchers and KBs participated, whereas only 20% of policymakers responded. This low response rate among policymakers may reflect limited availability or lower engagement with EBPM. Second, as the study focused specifically on maternal and child health, the generalizability of the findings to other policy domains may be limited. Third, this study assessed perceived effectiveness, rather than empirical effectiveness, and did not conduct inferential statistical testing. This approach was adopted because the interventions had not yet been implemented in real-world settings, and the primary aim was to explore the respondents’ perceptions rather than to confirm statistically significant differences. Therefore, perceived effectiveness was used as a proxy for practical value. Future research involving larger, more diverse sample sizes is needed to validate these descriptive trends through rigorous statistical analysis.

Despite these limitations, this study has several strengths. Notably, the clustering of perceived effectiveness scores between 3.91 and 4.53 indicates broad agreement on the relevance of the factors qualitatively identified in a previous study [14], thereby supporting both their validity and practical importance.

Furthermore, this study uniquely integrates qualitative factors with specific implementable interventions and systematically evaluates their perceived effectiveness. The prioritized interventions reflect practical considerations derived from stakeholder input, rather than theoretical assumptions, providing actionable insights for decision-makers seeking to allocate limited administrative, human and financial resources effectively to promote EBPM.

Finally, this study contributes to a better understanding of the Japanese policymaking context, which remains relatively under-researched in the global literature. In particular, it provides valuable insights into the roles of KBs and the unique personnel exchange system within the Japanese administrative framework.

### Conclusion and implications

This study identified the perceived effectiveness of 25 EBPM-promoting factors and 75 corresponding interventions on the basis of stakeholder input from policymakers, researchers and KBs. These findings provide a foundation for the implementation of strategies by presenting practical interventions. Descriptive trends in the perceived effectiveness across stakeholders indicate that implementation should reflect the priorities, roles and constraints of each group. Although this study focuses specifically on the Japanese policymaking context, EBPM in Japan remains relatively under-researched. Nevertheless, this study contributes to the academic literature on EBPM by systematically identifying the perceived effectiveness of the factors, linking each factor to specific interventions, and showing how stakeholder perspectives differ, thereby strengthening the empirical basis for developing strategies to promote EBPM in practice.

## Supplementary Information

Supplementary Material 1.

## Data Availability

The anonymized dataset supporting the findings of this study is available from the corresponding author, SS, upon reasonable request.

## Abbreviations

EBPM: Evidence-based policy making
KBs: Knowledge brokers
UK: United Kingdom
US: United States
