# Supplementary material for: Perceived effectiveness of factors and intervention strategies for promoting evidence-based policymaking in Japan: a cross-sectional survey among policymakers, researchers and knowledge brokers
Source: Health Res Policy Syst. 2026 Jul 11;24:85. doi: 10.1186/s12961-026-01501-5 (PMC13644055; doi:10.1186/s12961-026-01501-5)
Supplement: Supplementary file 1 [file 12961_2026_1501_MOESM1_ESM.docx]

Appendix: Factors with average and their corresponding interventions

| Ranking |  | Average | | | |  | Average (%) | | | |
| --- | --- | --- | --- | --- | --- | --- | --- | --- | --- | --- |
|  | Subcaetgories | All | Policymaker | Researcher | KBs | Intervention strategies | All | Policymaker | Researcher | KBs |
| 1 | Organising and disseminating leverageable evidence and data | 4.53 | 4.60 | 4.56 | 4.40 | Development of data infrastructure that researchers can easily utilise while ensuring considerations for personal information | 79.6 | 66.7 | 83.3 | 86.7 |
|  |  |  |  |  |  | Creation and dissemination of data by researchers that consider policy needs | 68.5 | 60.0 | 70.8 | 73.3 |
|  |  |  |  |  |  | Establishment of standardised policy evaluation indicators for local governments and corresponding data collection frameworks | 61.1 | 40.0 | 79.2 | 53.3 |
|  |  |  |  |  |  | Development of a data collection and publication system by ministries to enable policy impact evaluations by external organisations | 51.9 | 46.7 | 66.7 | 33.3 |
| 2 | Enhancing the knowledge and skills of policymakers and researchers in policymaking processes, EBPM and research | 4.37 | 4.40 | 4.33 | 4.40 | Development and provision of EBPM-related educational content for professional organisations (e.g., academic societies) and policymakers | 75.5 | 60.0 | 95.7 | 60.0 |
|  |  |  |  |  |  | Establishment of an in-ministry training system that enables policymakers to acquire essential skills regardless of their assigned department | 75.5 | 73.3 | 82.6 | 66.7 |
|  |  |  |  |  |  | Provision of training materials to new members of ministry committees to facilitate an understanding of administrative systems and policy processes (e.g., development and distribution of training materials by think tanks) | 56.6 | 40.0 | 60.9 | 66.7 |
| 3 | Ensuring appropriate allocation of budgetary, personnel and time resources within the government | 4.36 | 4.40 | 4.24 | 4.53 | Implementation of operational efficiency improvements through IT tools (e.g., adoption of online work platforms such as Teams and Slack) | 75.5 | 60.0 | 87.0 | 73.3 |
|  |  |  |  |  |  | Public disclosure of parliamentarians responsible for delayed parliamentary question notices | 58.5 | 80.0 | 26.1 | 86.7 |
|  |  |  |  |  |  | Relaxation of recruitment criteria to increase mid-career hires (e.g., removal of age and professional licence requirements) | 43.4 | 40.0 | 47.8 | 40.0 |
| 4 | Evaluating the current situation based on accurate data and qualitatively collected field insights | 4.35 | 4.33 | 4.48 | 4.13 | Establishment of forums for policy research experts and policymakers to regularly monitor domestic and international trends and practical realities | 77.8 | 80.0 | 79.2 | 73.3 |
|  |  |  |  |  |  | Establishment of a system within ministries to conduct high-quality research (e.g., implementation of surveys using validated measurement tools) | 68.5 | 60.0 | 70.8 | 73.3 |
|  |  |  |  |  |  | Establishment of external organisations capable of promptly collecting input from children, stakeholders, and frontline professionals | 51.9 | 46.7 | 75.0 | 20.0 |
| 4 | Developing flexible frameworks for adjusting policy trajectories | 4.35 | 4.27 | 4.52 | 4.13 | Establishment of platforms where policymakers and local government officials can discuss policies (e.g., creating opportunities for policymakers to understand implementation challenges and local government issues) | 88.7 | 92.9 | 91.7 | 80.0 |
| 6 | Continuing involvement in policymaking from external organisations with specialised knowledge and field expertise | 4.29 | 4.13 | 4.44 | 4.20 | Creation of a system that allows personnel exchange participants to remain engaged with the policies they worked on during their exchange period | 63.6 | 66.7 | 60.0 | 66.7 |
|  |  |  |  |  |  | Placement of researchers within ministries to conduct research on confidential administrative data | 50.9 | 26.7 | 68.0 | 46.7 |
|  |  |  |  |  |  | Public communication by external organisations on issues difficult for ministries to directly address (e.g., limitations of government administration) | 40.0 | 46.7 | 32.0 | 46.7 |
| 7 | Creating support systems to provide technical and psychological assistance to personnel exchange participants | 4.28 | 4.13 | 4.25 | 4.47 | Creating a guide to provide personnel exchange candidates and host organisations with the necessary knowledge prior to commencement | 87.0 | 86.7 | 87.5 | 86.7 |
| 8 | Establishing systems which can fairly reference evidence and expert opinions | 4.27 | 4.40 | 4.20 | 4.27 | Development of a system enabling policymakers to refer to scientific knowledge and academic research when assessing current conditions and setting policy agendas (e.g., access to PubMed from within ministries, creation of evidence databases organised by policy area) | 83.3 | 100.0 | 79.2 | 73.3 |
|  |  |  |  |  |  | Creation of a system allowing policymakers to consult experts when necessary (e.g., compilation of expert lists by the EBPM Promotion Office, development of mailing lists including both researchers and policymakers) | 81.5 | 73.3 | 95.8 | 66.7 |
| 9 | Improving the research environment in academia to encourage research that contributes to policies | 4.26 | 4.29 | 4.38 | 4.07 | Enhancement of public statistical information infrastructure to facilitate its utilisation by academia | 86.3 | 92.3 | 87.5 | 78.6 |
|  |  |  |  |  |  | Expansion of research funding | 80.4 | 53.8 | 100.0 | 71.4 |
|  |  |  |  |  |  | Establishment of networks among researchers | 62.7 | 46.2 | 79.2 | 50.0 |
|  |  |  |  |  |  | Establishment of a researcher evaluation system that recognises publications related to policy impact assessment | 41.2 | 38.5 | 45.8 | 35.7 |
| 10 | Generating personnel exchange participants by providing lectures and hands-on experience and establishing human resource development systems | 4.23 | 4.07 | 4.21 | 4.40 | Development of a system allowing personnel exchange participants to engage in bidirectional interactions with government agencies, academia, and clinical settings to learn from diverse perspectives and roles | 82.7 | 78.6 | 87.0 | 80.0 |
|  |  |  |  |  |  | Strengthening of public health workforce development through the enhancement of healthcare policy and public health curricula in public health graduate schools and medical education | 73.1 | 71.4 | 78.3 | 66.7 |
|  |  |  |  |  |  | Provision of practical support for personnel exchange participants by external organisations (e.g., consulting firms) | 42.3 | 35.7 | 52.2 | 33.3 |
| 11 | Introducing schemes to strengthen implementation capabilities at the local government level | 4.22 | 4.29 | 4.32 | 4.00 | Strengthening of systems enabling local governments to share and reference best practices (e.g., holding local government best practice presentation sessions, creating online platforms to access exemplary cases and tools) | 73.1 | 92.3 | 75.0 | 53.3 |
|  |  |  |  |  |  | Development of training materials by external organisations to enhance EBPM-related skills among professionals such as public health nurses and midwives (e.g., creation and dissemination of training resources in collaboration with professional associations) | 51.9 | 46.2 | 58.3 | 46.7 |
|  |  |  |  |  |  | Facilitation by external organisations to connect local governments with private sector and academic institutions based on their policy needs | 51.9 | 46.2 | 66.7 | 33.3 |
|  |  |  |  |  |  | Design and implementation of model projects | 50.0 | 53.8 | 54.2 | 40.0 |
|  |  |  |  |  |  | Support for effective and efficient policy implementation by external organisations (e.g., consulting firms) | 46.2 | 69.2 | 41.7 | 33.3 |
|  |  |  |  |  |  | Implementation of industry-government-academia-civil society collaboration support programmes (e.g., incubation programmes to connect social enterprises with public and private funding sources) | 36.5 | 38.5 | 50.0 | 13.3 |
|  |  |  |  |  |  | Government support for the establishment of EBPM Promotion Offices within local governments (e.g., implementation of training programmes for local policymakers on EBPM) | 34.6 | 30.8 | 45.8 | 20.0 |
| 11 | Providing organisational support to utilise the experiences of personnel exchange participants | 4.22 | 4.07 | 4.17 | 4.47 | Establishment of a personnel system in the dispatching organisations to enable personnel exchange participants to leverage their administrative experience post-exchange | 75.9 | 86.7 | 66.7 | 80.0 |
|  |  |  |  |  |  | Establishment of platforms where personnel exchange participants can act as bridges between academia and policy (e.g., creation of policy committees) | 72.2 | 66.7 | 83.3 | 60.0 |
|  |  |  |  |  |  | Provision of posts where personnel exchange participants can apply their experiences | 70.4 | 86.7 | 58.3 | 73.3 |
|  |  |  |  |  |  | Development of a system where personnel exchange participants can provide insights to researchers during policy-related research agenda setting | 55.6 | 46.7 | 66.7 | 46.7 |
| 11 | Promoting shared inter-organisational awareness throughout the EBPM and at each stage | 4.22 | 4.13 | 4.36 | 4.07 | Distribution of training materials by external organisations to facilitate learning on EBPM, policy processes, and research fundamentals for both policymakers and researchers (e.g., development of training materials by think tanks to establish shared stakeholder understanding of EBPM and evidence dissemination methods) | 71.7 | 66.7 | 78.3 | 66.7 |
| 14 | Setting agendas from a comprehensive perspective | 4.21 | 4.08 | 4.32 | 4.14 | Establishment of platforms for representatives of academic societies and academia to engage in discussions with policymakers on current policy challenges | 90.2 | 84.6 | 91.7 | 92.9 |
|  |  |  |  |  |  | Establishment of a system to reflect the outcomes of discussions with representatives of academic societies and academia in policy | 66.7 | 30.8 | 75.0 | 85.7 |
|  |  |  |  |  |  | Creation of forums facilitated by external organisations for policymakers to prioritise policy challenges while considering diverse opinions (e.g., workshops conducted by think tanks involving policymakers, researchers, and stakeholders) | 60.8 | 38.5 | 66.7 | 71.4 |
|  |  |  |  |  |  | Establishment of methodologies within expert organisations (e.g., academic societies) to determine policy priority-setting mechanisms | 45.1 | 23.1 | 66.7 | 28.6 |
| 15 | Implementing administrative procedures that reflect not only central government ministries and agencies but also external organisational perspectives in policy evaluation and effectively utilise the outcomes | 4.20 | 4.00 | 4.44 | 4.00 | Establishment of forums for regular discussions between local governments, external organisations, and policymakers on individual policy evaluations | 69.8 | 64.3 | 83.3 | 53.3 |
|  |  |  |  |  |  | Implementation of policy evaluations in collaboration with local governments and external organisations post-policy implementation | 62.3 | 57.1 | 70.8 | 53.3 |
|  |  |  |  |  |  | Creation of a system that allows policymakers to easily reference policy evaluations conducted by external organisations | 56.6 | 42.9 | 66.7 | 53.3 |
| 15 | Introducing career opportunities for personnel exchange participants after their exchange programmes | 4.20 | 4.20 | 4.17 | 4.27 | Expansion and facilitation of external posts where policymakers can utilise their skills beyond administrative roles | 68.0 | 61.5 | 77.3 | 60.0 |
|  |  |  |  |  |  | Creation of an online platform by external organisations where personnel exchange participants can share experiences and career paths | 52.0 | 53.8 | 59.1 | 40.0 |
| 15 | Ensuring the thorough dissemination of policies and information to local governments and policy beneficiaries | 4.20 | 4.00 | 4.28 | 4.27 | Development of an information dissemination system by ministries that takes policy beneficiaries into consideration (e.g., dissemination of administrative information through effective media for different generations) | 78.8 | 61.5 | 91.7 | 73.3 |
|  |  |  |  |  |  | Development of a support system by external organisations to connect researcher-generated materials and research findings to policy (e.g., think tanks providing tailored support to deliver materials to policy beneficiaries) | 73.1 | 84.6 | 66.7 | 73.3 |
|  |  |  |  |  |  | Development of an appropriate media relations framework by ministries | 50.0 | 46.2 | 66.7 | 26.7 |
| 18 | Establishing and utilising think tanks to provide scientifically reliable information | 4.19 | 4.00 | 4.25 | 4.27 | Public disclosure of aggregated data in an accessible manner (e.g., creation of databases that are easy to understand for policymakers, policy beneficiaries, and researchers; mapping of domestic and international information) | 73.6 | 78.6 | 66.7 | 80.0 |
|  |  |  |  |  |  | Timely data aggregation | 66.0 | 57.1 | 70.8 | 66.7 |
|  |  |  |  |  |  | Development of a support system for personnel exchange participants (e.g., creation of networking events and mailing lists for past participants) | 66.0 | 57.1 | 62.5 | 80.0 |
|  |  |  |  |  |  | Establishment of a system for personnel exchanges between researchers and policymakers | 54.7 | 71.4 | 50.0 | 46.7 |
|  |  |  |  |  |  | Establishment and operation of specialised think tanks | 50.9 | 35.7 | 62.5 | 46.7 |
|  |  |  |  |  |  | Continuous participation and recommendations in policy decision-making forums (e.g., involvement in councils for maternal and child health) | 47.2 | 28.6 | 62.5 | 40.0 |
|  |  |  |  |  |  | Implementation of early-stage policy engagement and ongoing support | 45.3 | 42.9 | 50.0 | 40.0 |
|  |  |  |  |  |  | Collaboration with international organisations (e.g., WHO) to conduct comparative studies and generate evidence | 43.4 | 28.6 | 58.3 | 33.3 |
| 19 | Creating platforms for active exchange of opinions and relationships-building across organisational boundaries | 4.18 | 3.93 | 4.40 | 4.07 | Creation of closed forums facilitated by external organisations for policymakers and researchers to exchange information (e.g., establishment of confidential discussion spaces by think tanks) | 80.8 | 78.6 | 87.0 | 73.3 |
| 19 | Generating flexible evidence by researchers meeting the policy needs and establishing administrative mechanisms to leverage them | 4.18 | 4.00 | 4.36 | 4.07 | Establishment of platforms to match researchers with policy needs and policymakers with policy seeds | 80.0 | 66.7 | 84.0 | 86.7 |
|  |  |  |  |  |  | Establishment and operation of external organisations focused on policy-relevant research | 49.1 | 40.0 | 56.0 | 46.7 |
|  |  |  |  |  |  | Deployment of policy research experts in each policy area to provide inputs during policy design | 45.5 | 33.3 | 56.0 | 40.0 |
| 21 | Citizen participatory policy formation | 4.15 | 4.00 | 4.24 | 4.13 | Establishment of an in-ministry system that ensures policy beneficiaries and healthcare/public health professionals can continuously participate in policy decision-making forums (e.g., councils and advisory committees) | 76.5 | 61.5 | 82.6 | 80.0 |
|  |  |  |  |  |  | Implementation of policy evaluations within ministries that incorporate the perspectives of policy beneficiaries (e.g., inclusion of policy beneficiaries in ministry policy evaluation discussions) | 62.7 | 53.8 | 82.6 | 40.0 |
| 21 | Increasing awareness and establishing evaluation systems for policies within the government | 4.15 | 3.93 | 4.40 | 3.93 | Strengthening of systems that enable policymakers to acquire knowledge of Evidence-Based Policy Making (EBPM) and policy processes, as well as improve their practical skills | 78.8 | 92.9 | 66.7 | 85.7 |
|  |  |  |  |  |  | Distribution of workshops and training materials to establish a flexible and responsive policy-making cycle (e.g., continuous provision of EBPM learning content) | 51.9 | 35.7 | 66.7 | 42.9 |
|  |  |  |  |  |  | Strengthening of the EBPM Promotion Office’s policy oversight functions (e.g., enhanced collaboration with external policy evaluation organisations) | 42.3 | 50.0 | 45.8 | 28.6 |
| 23 | Constructing personnel evaluation systems considering their impact on policies in academia | 4 | 3.77 | 4.20 | 3.86 | Development of a research personnel evaluation system that considers social impact in addition to research output (e.g., number of lead researchers in health policy research, implementation research in local governments) | 68.8 | 66.7 | 73.9 | 61.5 |
|  |  |  |  |  |  | Organisation of criteria to evaluate researchers' policy engagement experience | 60.4 | 50.0 | 69.6 | 53.8 |
| 24 | Instituting administrative personnel systems that enable long-term engagement in policies | 3.98 | 3.53 | 4.28 | 3.93 | Development of a personnel system enabling policymakers to engage in policy within their specialised fields over the long term (e.g., a system allowing officials to plan career paths aligned with their expertise) | 77.4 | 64.3 | 79.2 | 86.7 |
|  |  |  |  |  |  | Development of a ministry system that ensures continuity of operations irrespective of policymakers’ terms (e.g., utilisation of IT tools to continuously update work records and ensure smooth handovers) | 66.0 | 35.7 | 79.2 | 73.3 |
| 25 | Establishing personnel evaluation systems considering the policy implementation performance of policymakers and policy evaluations | 3.91 | 3.60 | 3.92 | 4.20 | Clarification of the competencies required for policymakers in each policy area | 62.7 | 41.7 | 66.7 | 73.3 |
|  |  |  |  |  |  | Development of a personnel evaluation system that assesses the execution capabilities of policymakers | 60.8 | 41.7 | 66.7 | 66.7 |
|  |  |  |  |  |  | Implementation of personnel evaluation for policymakers in collaboration with external organisations | 39.2 | 25.0 | 50.0 | 33.3 |
|  |  |  |  |  |  |  |  |  |  |  |
